# Supplementary figures and images for: Automatic spread factor and position definition for UAV gateway through computational intelligence approach to maximize signal-to-noise ratio in wooded environments
Source: PeerJ Comput Sci. 2024 Sep 27;10:e2237. doi: 10.7717/peerj-cs.2237 (PMC11622905; doi:10.7717/peerj-cs.2237)

# Pareto: 1

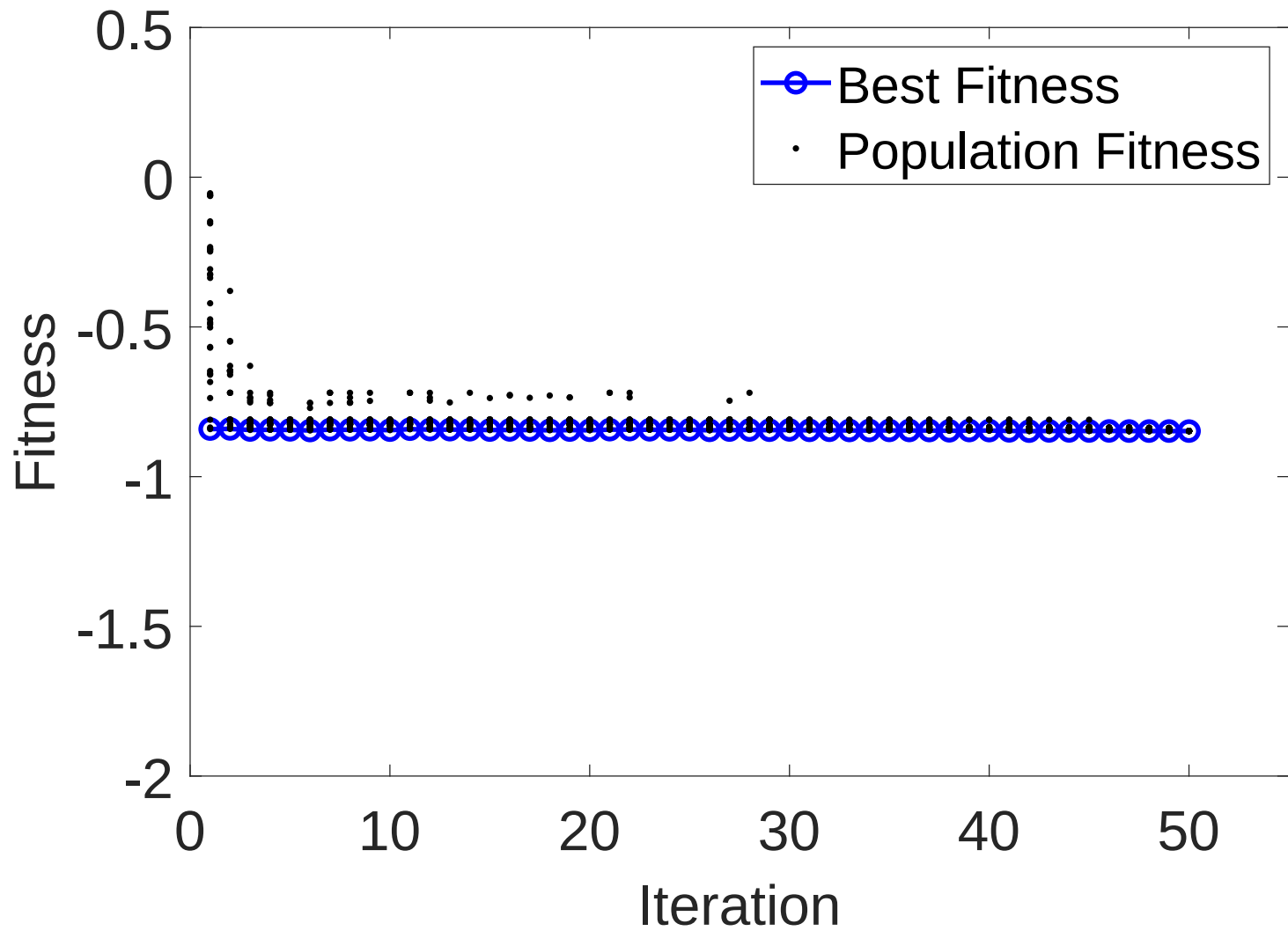

Supplement: Supplemental Information 1 — The code (otimizador_ufpa) to run the adapted gwo optimizer for the UFPA scenario. - code "main.m" runs the optimizer - code "trata_resultados.m" plots the general analysis for all paretos - folder "resultados" contains the plots used in the draft and the results - obtained for the optimizer in .mat files The code (SNR_mlp) to train the grnn and mlp networks: - "old_mlp_snr_v2.m" trains the mlp networks - "grnn_snr.m" trains the grnn networks - "analise_resultados.m" generates the boxplot to compare grnn x mlp, then find the best network [file peerj-cs-10-2237-s001.zip › peerJ/otimizador_ufpa/resultados/convergencia_0_pareto_1_fitness_atualizada.pdf]

# Pareto: 10

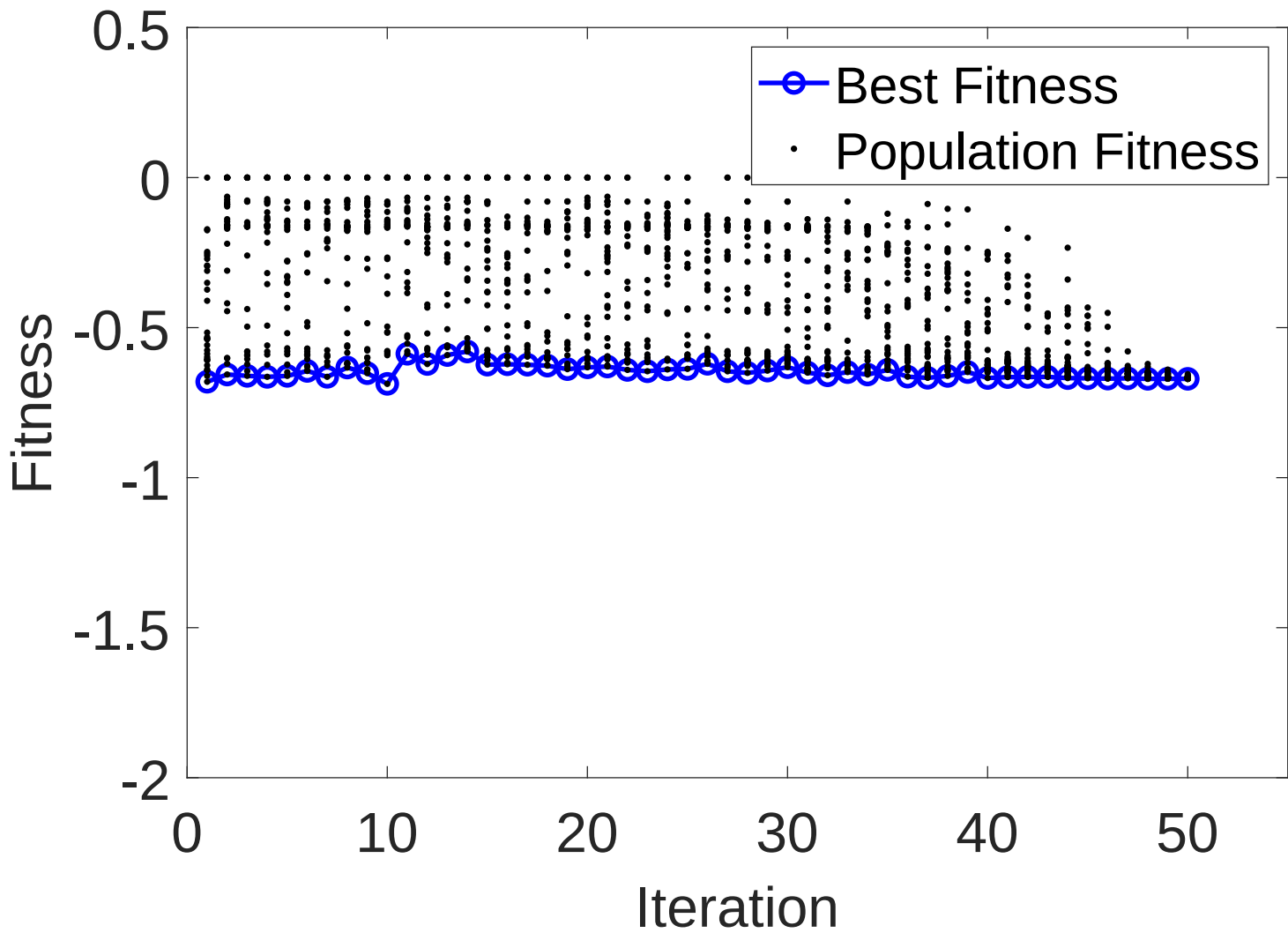

Supplement: Supplemental Information 1 — The code (otimizador_ufpa) to run the adapted gwo optimizer for the UFPA scenario. - code "main.m" runs the optimizer - code "trata_resultados.m" plots the general analysis for all paretos - folder "resultados" contains the plots used in the draft and the results - obtained for the optimizer in .mat files The code (SNR_mlp) to train the grnn and mlp networks: - "old_mlp_snr_v2.m" trains the mlp networks - "grnn_snr.m" trains the grnn networks - "analise_resultados.m" generates the boxplot to compare grnn x mlp, then find the best network [file peerj-cs-10-2237-s001.zip › peerJ/otimizador_ufpa/resultados/convergencia_0_pareto_10_fitness_atualizada.pdf]

## Pareto: 2

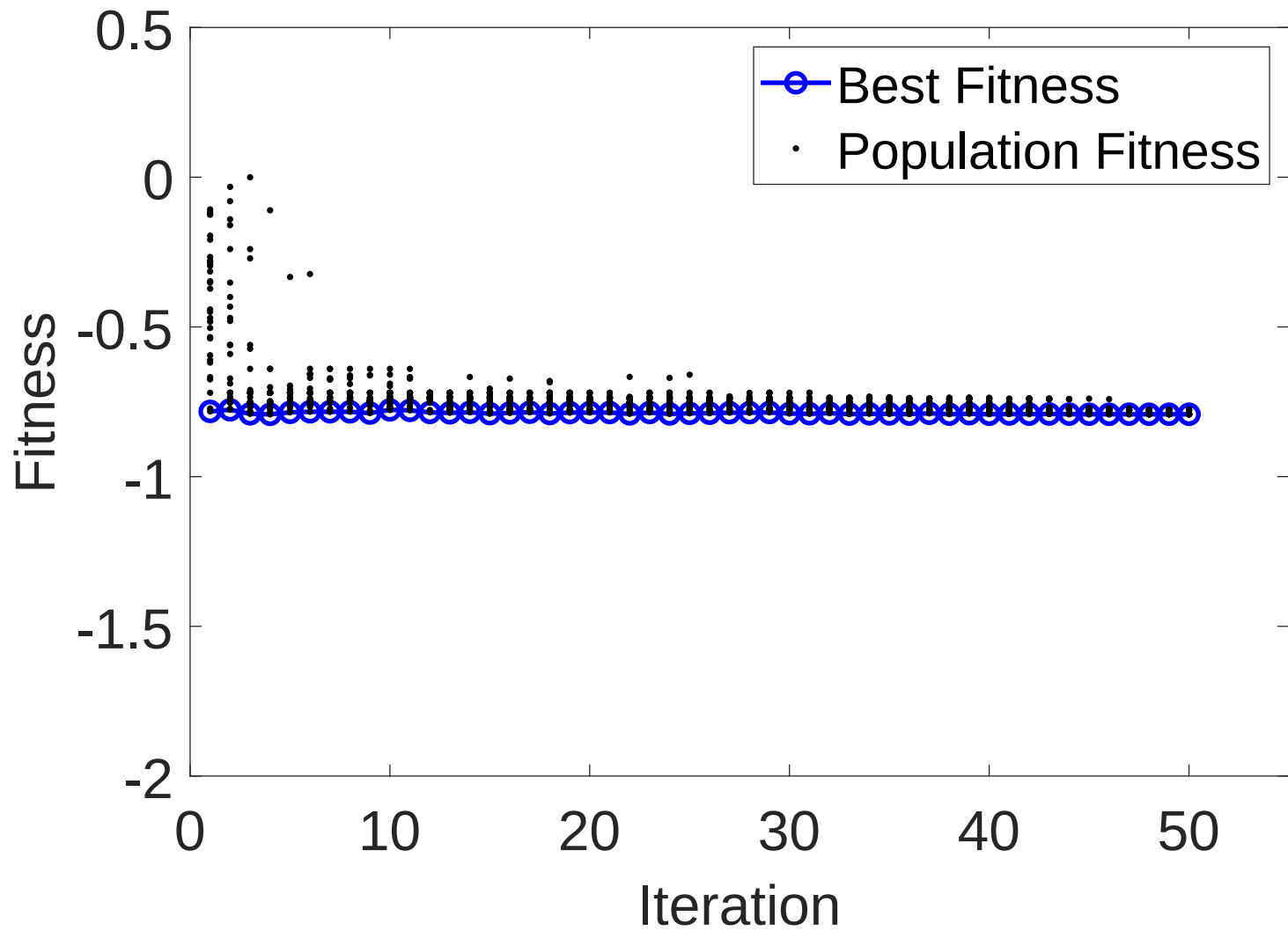

Supplement: Supplemental Information 1 — The code (otimizador_ufpa) to run the adapted gwo optimizer for the UFPA scenario. - code "main.m" runs the optimizer - code "trata_resultados.m" plots the general analysis for all paretos - folder "resultados" contains the plots used in the draft and the results - obtained for the optimizer in .mat files The code (SNR_mlp) to train the grnn and mlp networks: - "old_mlp_snr_v2.m" trains the mlp networks - "grnn_snr.m" trains the grnn networks - "analise_resultados.m" generates the boxplot to compare grnn x mlp, then find the best network [file peerj-cs-10-2237-s001.zip › peerJ/otimizador_ufpa/resultados/convergencia_0_pareto_2_fitness_atualizada.pdf]

# Pareto: 3

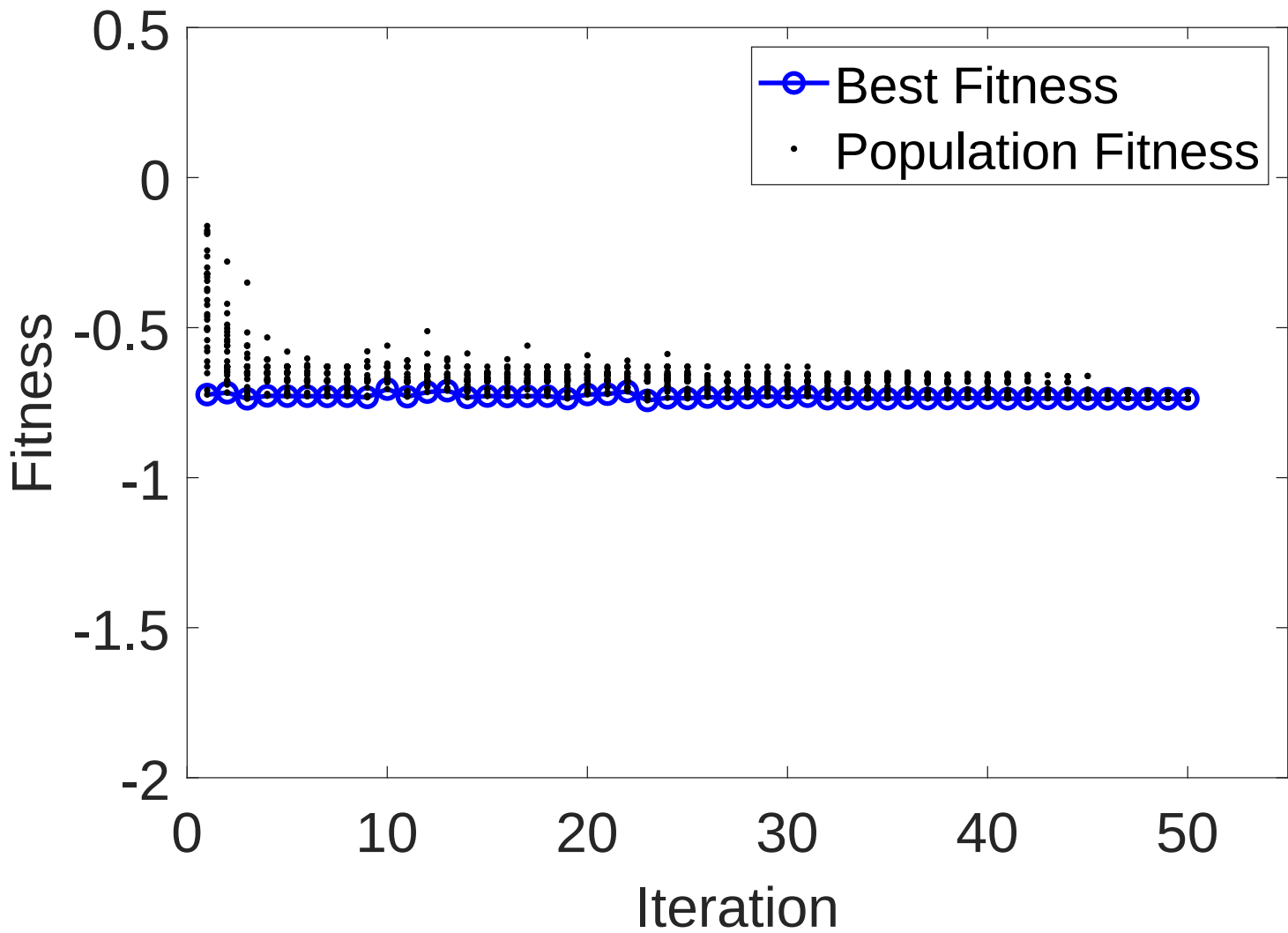

Supplement: Supplemental Information 1 — The code (otimizador_ufpa) to run the adapted gwo optimizer for the UFPA scenario. - code "main.m" runs the optimizer - code "trata_resultados.m" plots the general analysis for all paretos - folder "resultados" contains the plots used in the draft and the results - obtained for the optimizer in .mat files The code (SNR_mlp) to train the grnn and mlp networks: - "old_mlp_snr_v2.m" trains the mlp networks - "grnn_snr.m" trains the grnn networks - "analise_resultados.m" generates the boxplot to compare grnn x mlp, then find the best network [file peerj-cs-10-2237-s001.zip › peerJ/otimizador_ufpa/resultados/convergencia_0_pareto_3_fitness_atualizada.pdf]

# Pareto: 4

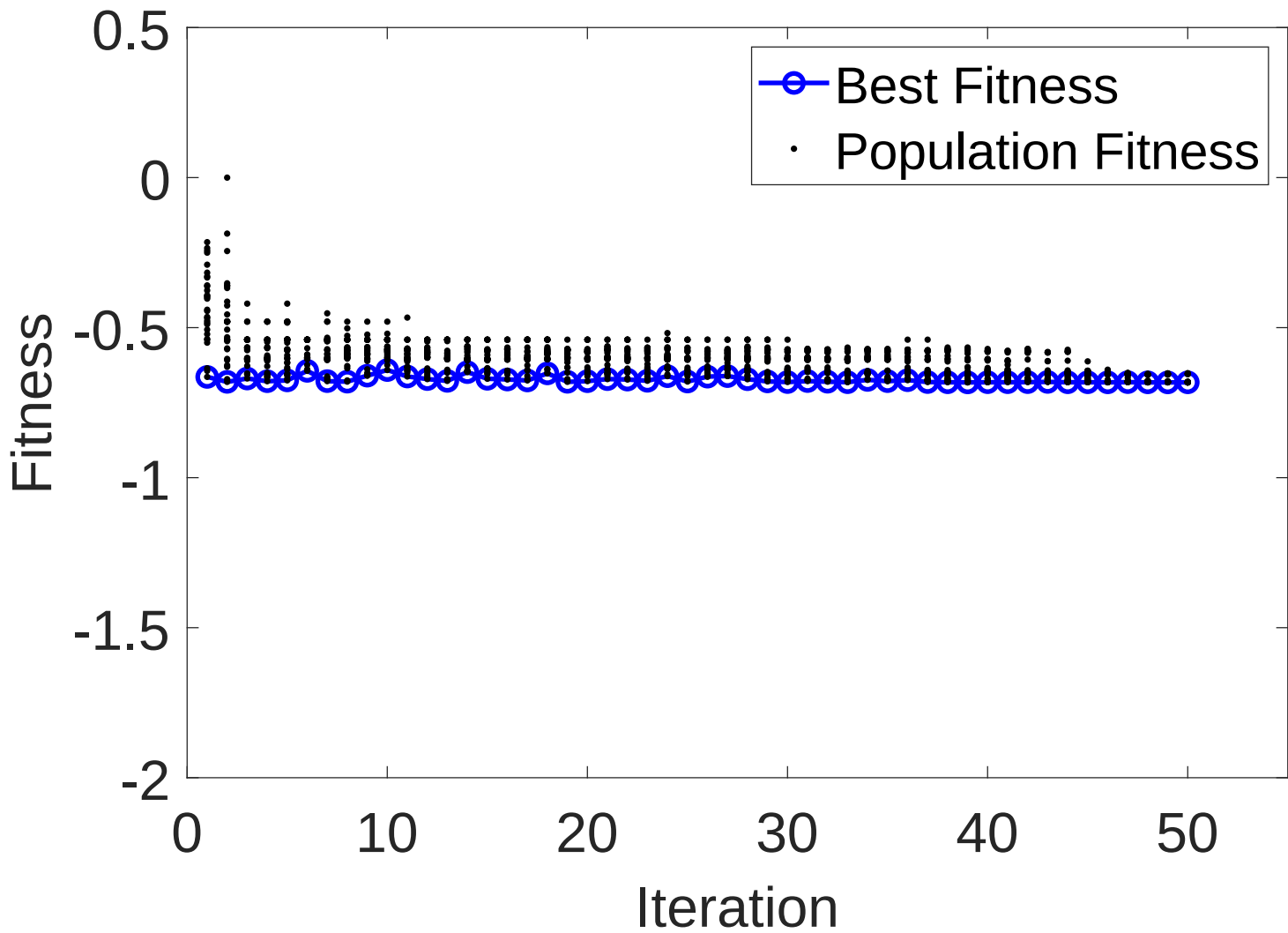

Supplement: Supplemental Information 1 — The code (otimizador_ufpa) to run the adapted gwo optimizer for the UFPA scenario. - code "main.m" runs the optimizer - code "trata_resultados.m" plots the general analysis for all paretos - folder "resultados" contains the plots used in the draft and the results - obtained for the optimizer in .mat files The code (SNR_mlp) to train the grnn and mlp networks: - "old_mlp_snr_v2.m" trains the mlp networks - "grnn_snr.m" trains the grnn networks - "analise_resultados.m" generates the boxplot to compare grnn x mlp, then find the best network [file peerj-cs-10-2237-s001.zip › peerJ/otimizador_ufpa/resultados/convergencia_0_pareto_4_fitness_atualizada.pdf]

# Pareto: 5

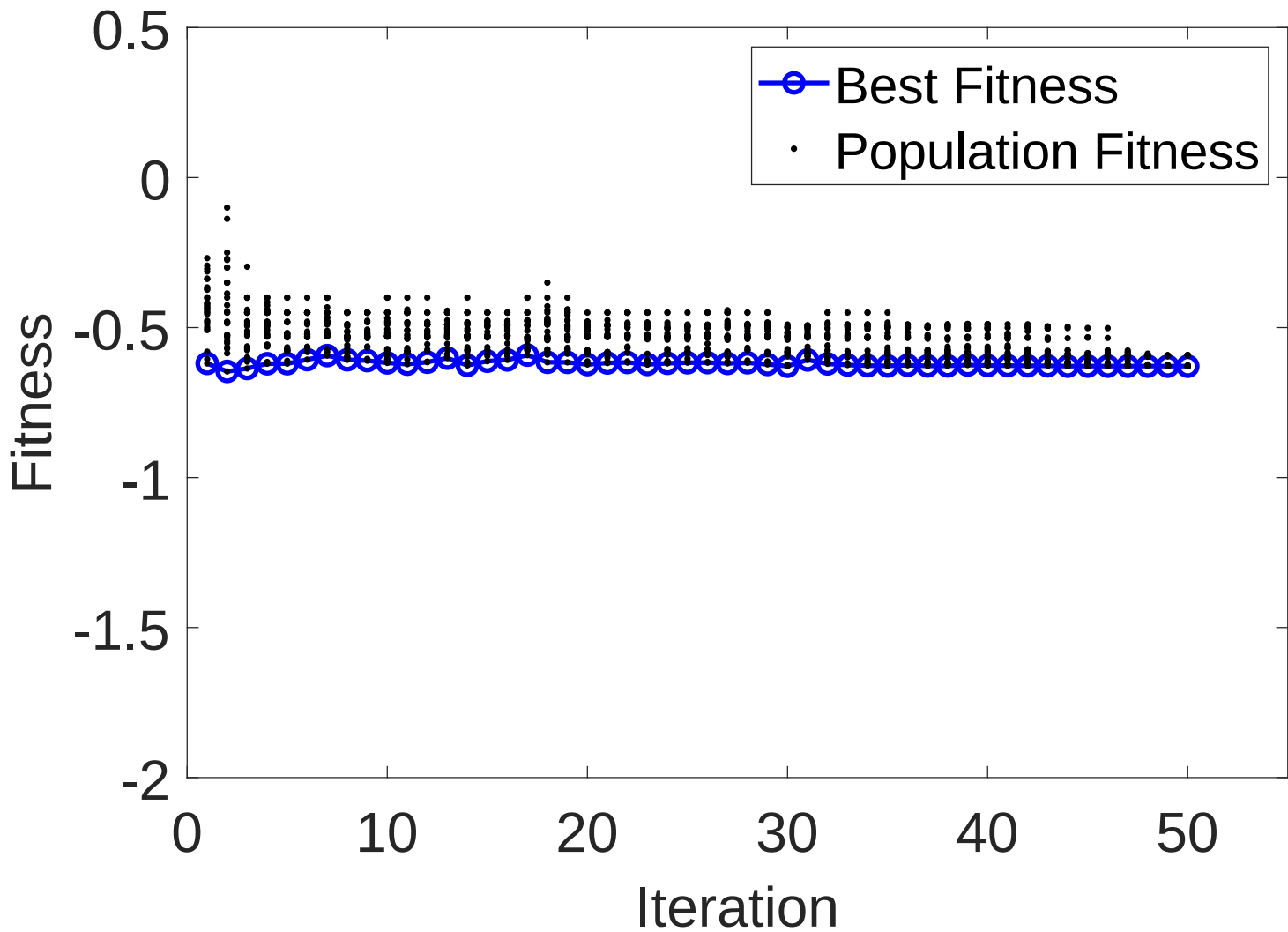

Supplement: Supplemental Information 1 — The code (otimizador_ufpa) to run the adapted gwo optimizer for the UFPA scenario. - code "main.m" runs the optimizer - code "trata_resultados.m" plots the general analysis for all paretos - folder "resultados" contains the plots used in the draft and the results - obtained for the optimizer in .mat files The code (SNR_mlp) to train the grnn and mlp networks: - "old_mlp_snr_v2.m" trains the mlp networks - "grnn_snr.m" trains the grnn networks - "analise_resultados.m" generates the boxplot to compare grnn x mlp, then find the best network [file peerj-cs-10-2237-s001.zip › peerJ/otimizador_ufpa/resultados/convergencia_0_pareto_5_fitness_atualizada.pdf]

# Pareto: 6

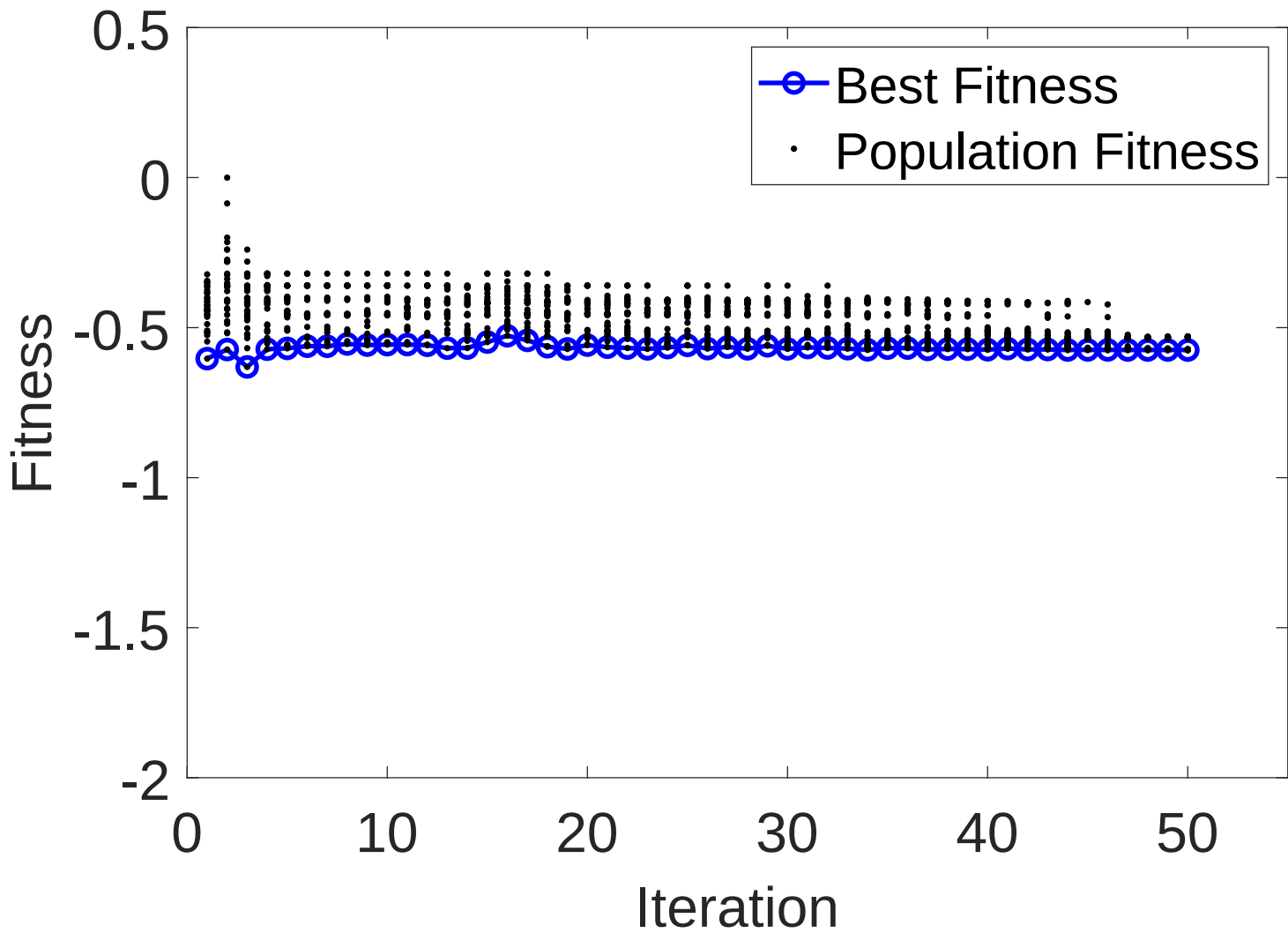

Supplement: Supplemental Information 1 — The code (otimizador_ufpa) to run the adapted gwo optimizer for the UFPA scenario. - code "main.m" runs the optimizer - code "trata_resultados.m" plots the general analysis for all paretos - folder "resultados" contains the plots used in the draft and the results - obtained for the optimizer in .mat files The code (SNR_mlp) to train the grnn and mlp networks: - "old_mlp_snr_v2.m" trains the mlp networks - "grnn_snr.m" trains the grnn networks - "analise_resultados.m" generates the boxplot to compare grnn x mlp, then find the best network [file peerj-cs-10-2237-s001.zip › peerJ/otimizador_ufpa/resultados/convergencia_0_pareto_6_fitness_atualizada.pdf]

# Pareto: 7

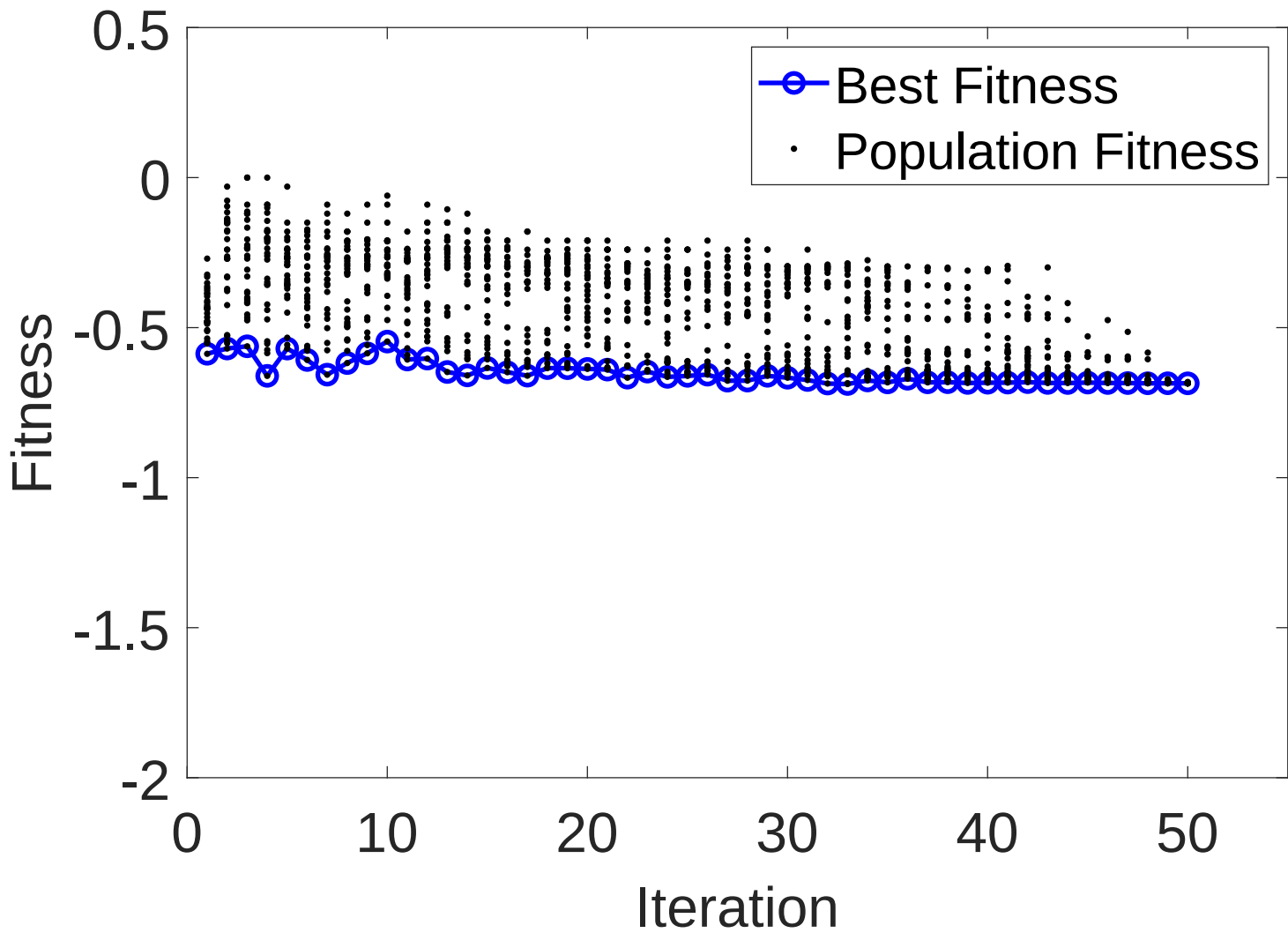

Supplement: Supplemental Information 1 — The code (otimizador_ufpa) to run the adapted gwo optimizer for the UFPA scenario. - code "main.m" runs the optimizer - code "trata_resultados.m" plots the general analysis for all paretos - folder "resultados" contains the plots used in the draft and the results - obtained for the optimizer in .mat files The code (SNR_mlp) to train the grnn and mlp networks: - "old_mlp_snr_v2.m" trains the mlp networks - "grnn_snr.m" trains the grnn networks - "analise_resultados.m" generates the boxplot to compare grnn x mlp, then find the best network [file peerj-cs-10-2237-s001.zip › peerJ/otimizador_ufpa/resultados/convergencia_0_pareto_7_fitness_atualizada.pdf]

# Pareto: 8

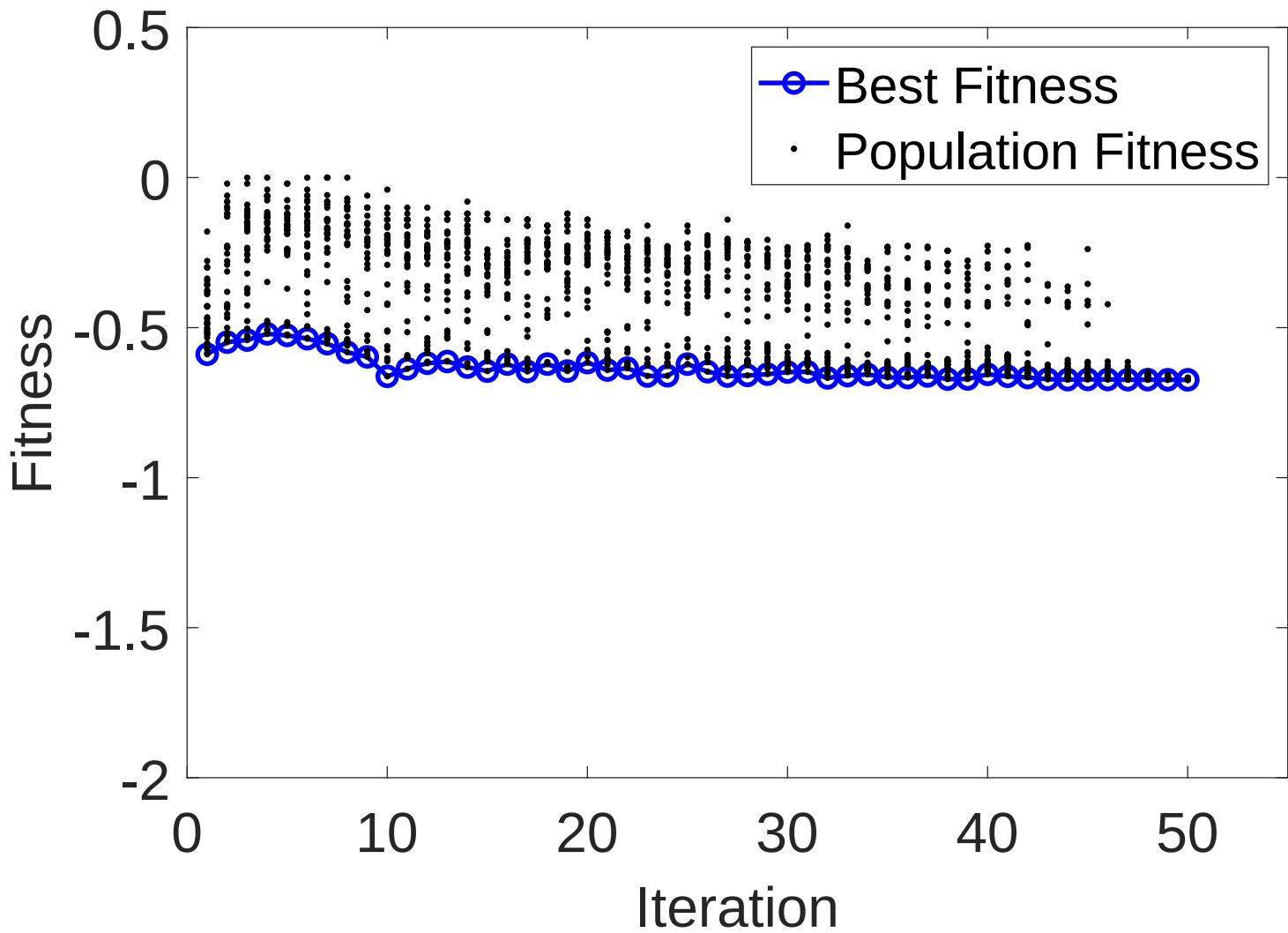

Supplement: Supplemental Information 1 — The code (otimizador_ufpa) to run the adapted gwo optimizer for the UFPA scenario. - code "main.m" runs the optimizer - code "trata_resultados.m" plots the general analysis for all paretos - folder "resultados" contains the plots used in the draft and the results - obtained for the optimizer in .mat files The code (SNR_mlp) to train the grnn and mlp networks: - "old_mlp_snr_v2.m" trains the mlp networks - "grnn_snr.m" trains the grnn networks - "analise_resultados.m" generates the boxplot to compare grnn x mlp, then find the best network [file peerj-cs-10-2237-s001.zip › peerJ/otimizador_ufpa/resultados/convergencia_0_pareto_8_fitness_atualizada.pdf]

# Pareto: 9

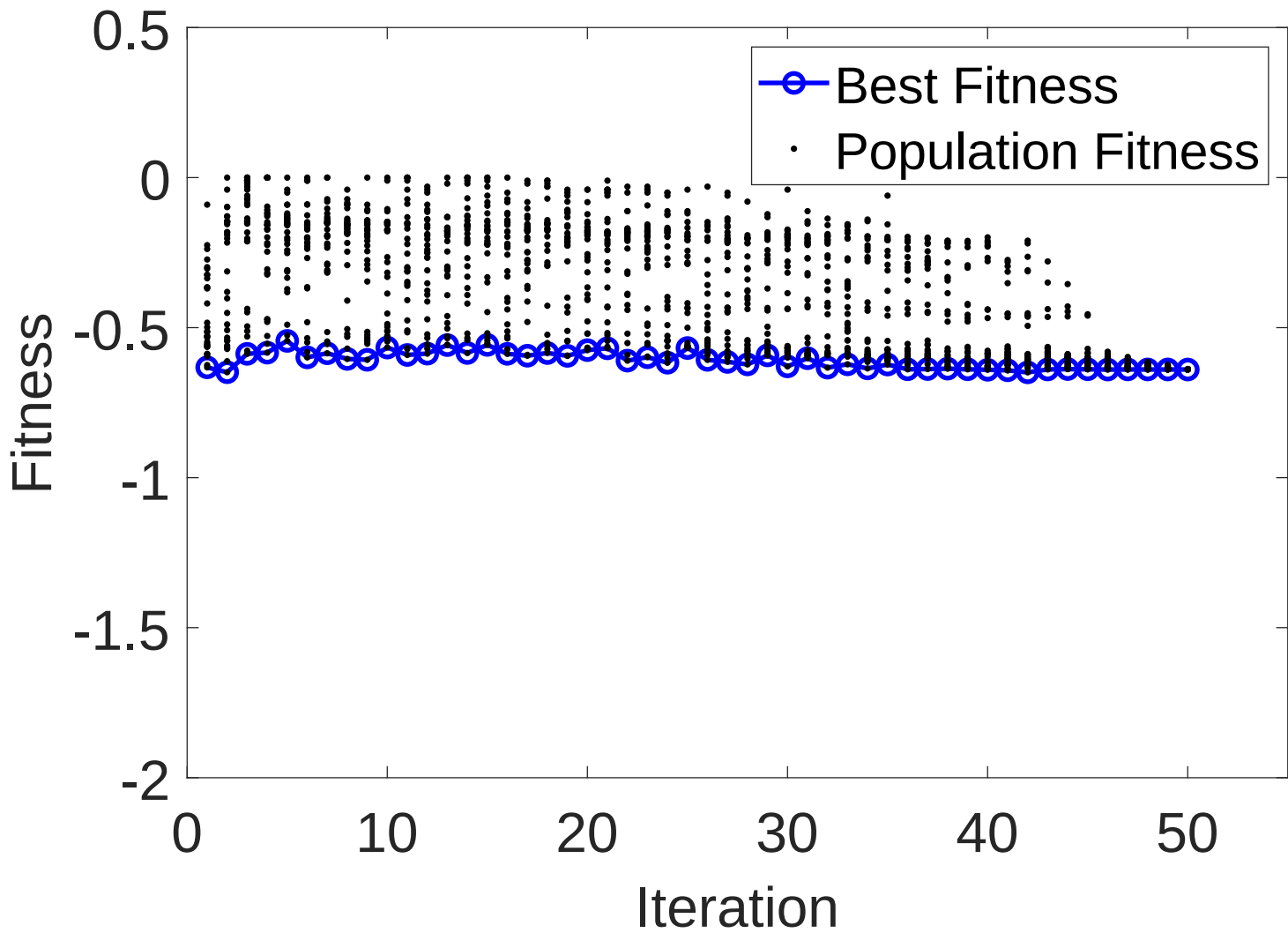

Supplement: Supplemental Information 1 — The code (otimizador_ufpa) to run the adapted gwo optimizer for the UFPA scenario. - code "main.m" runs the optimizer - code "trata_resultados.m" plots the general analysis for all paretos - folder "resultados" contains the plots used in the draft and the results - obtained for the optimizer in .mat files The code (SNR_mlp) to train the grnn and mlp networks: - "old_mlp_snr_v2.m" trains the mlp networks - "grnn_snr.m" trains the grnn networks - "analise_resultados.m" generates the boxplot to compare grnn x mlp, then find the best network [file peerj-cs-10-2237-s001.zip › peerJ/otimizador_ufpa/resultados/convergencia_0_pareto_9_fitness_atualizada.pdf]

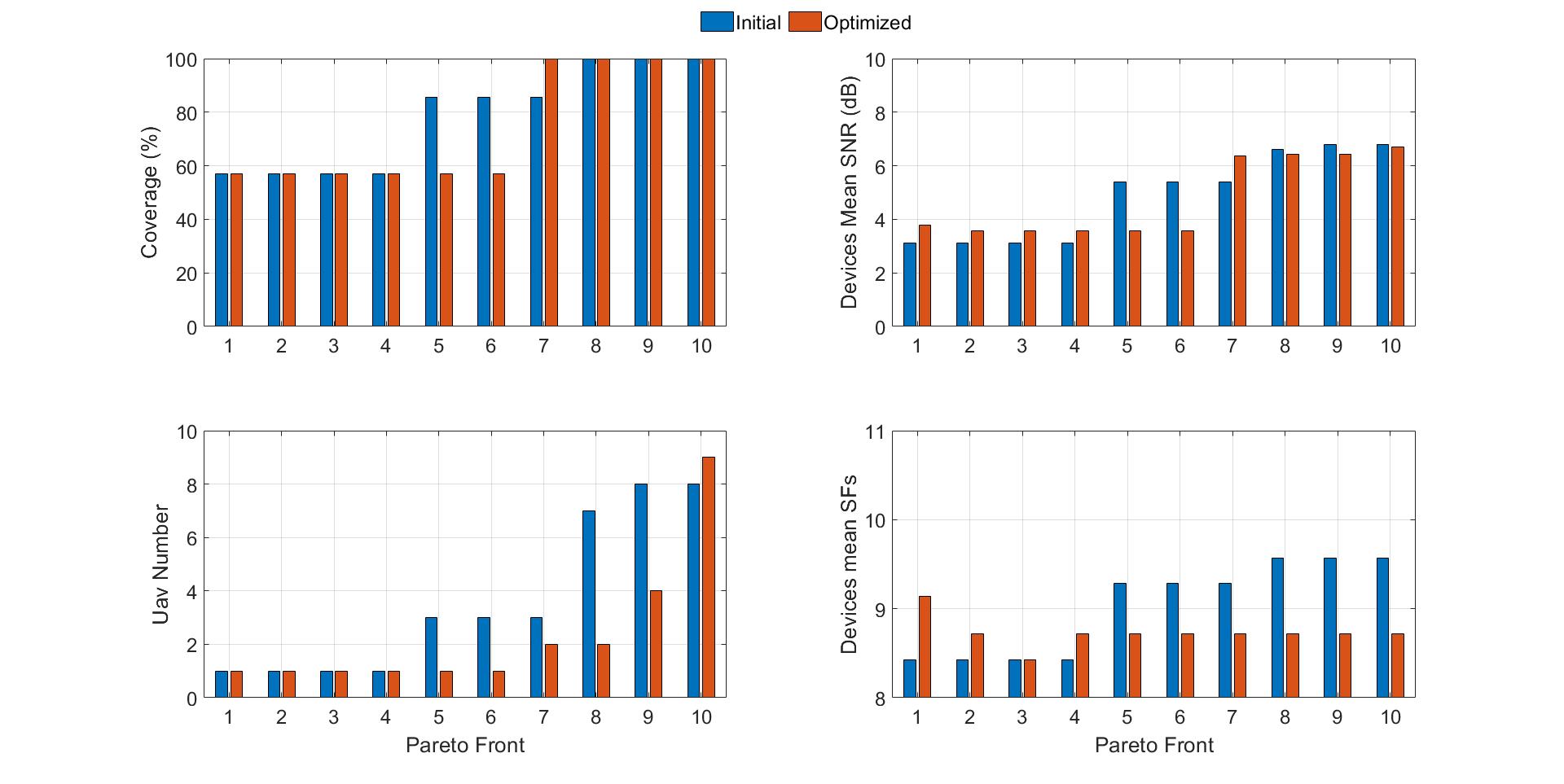

Supplement: Supplemental Information 1 — The code (otimizador_ufpa) to run the adapted gwo optimizer for the UFPA scenario. - code "main.m" runs the optimizer - code "trata_resultados.m" plots the general analysis for all paretos - folder "resultados" contains the plots used in the draft and the results - obtained for the optimizer in .mat files The code (SNR_mlp) to train the grnn and mlp networks: - "old_mlp_snr_v2.m" trains the mlp networks - "grnn_snr.m" trains the grnn networks - "analise_resultados.m" generates the boxplot to compare grnn x mlp, then find the best network [file peerj-cs-10-2237-s001.zip › peerJ/otimizador_ufpa/resultados/resultados_para_todos_paretos.jpg]

## UAV Optimization Pareto: 2

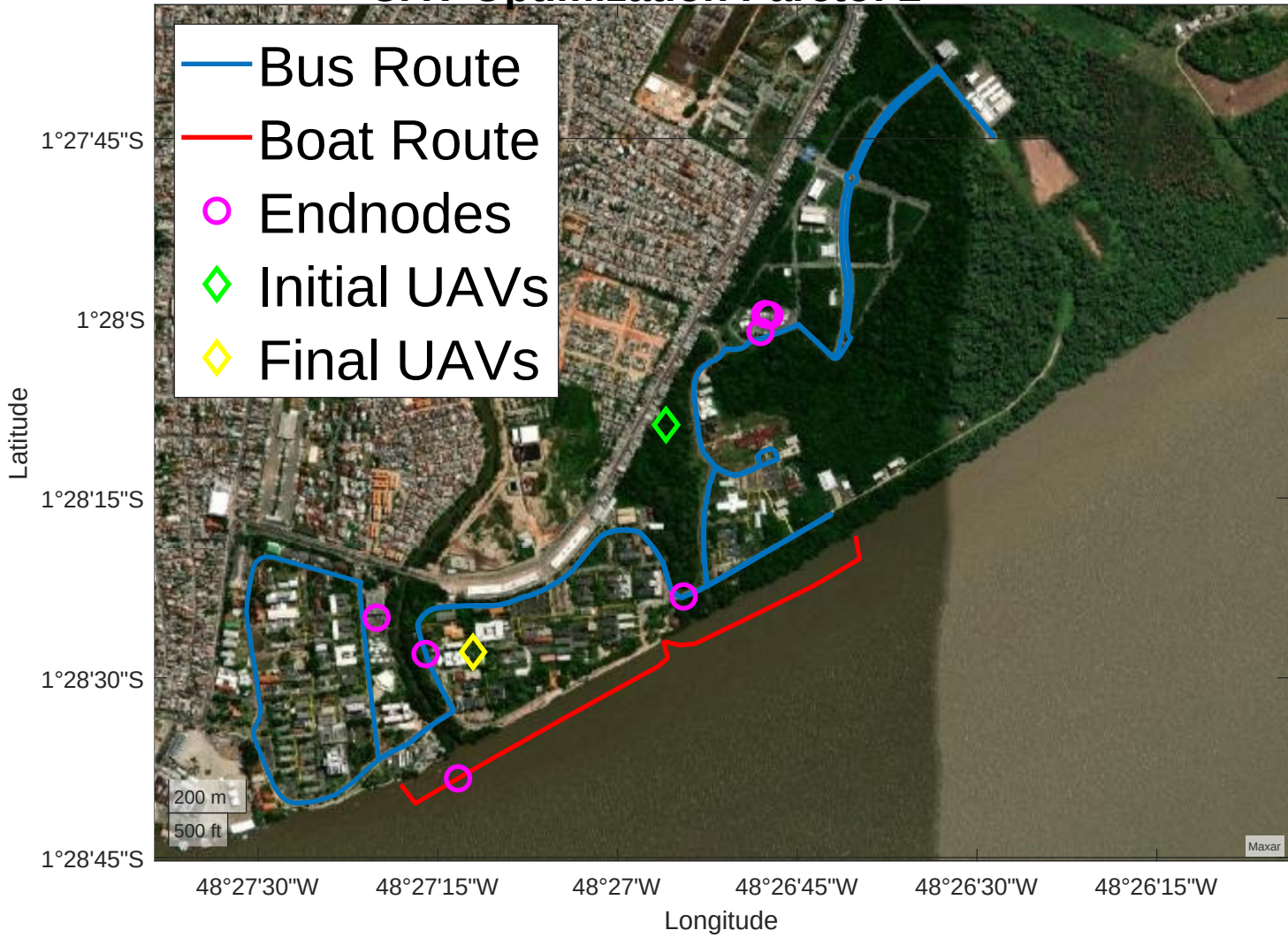

Supplement: Supplemental Information 11 [file peerj-cs-10-2237-s011.pdf]

# UAV Optimization Pareto: 3

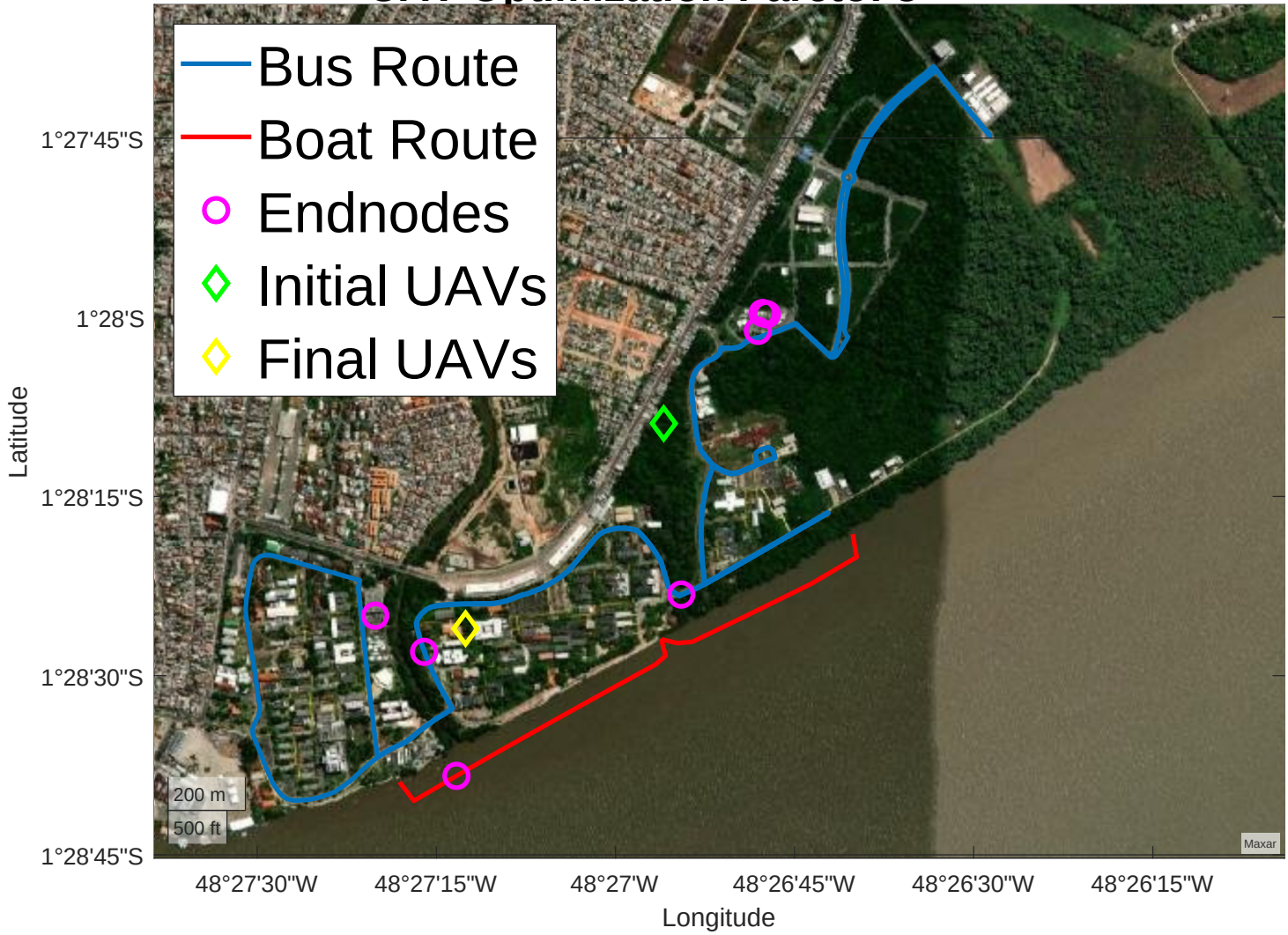

Supplement: Supplemental Information 12 [file peerj-cs-10-2237-s012.pdf]

# UAV Optimization Pareto: 4

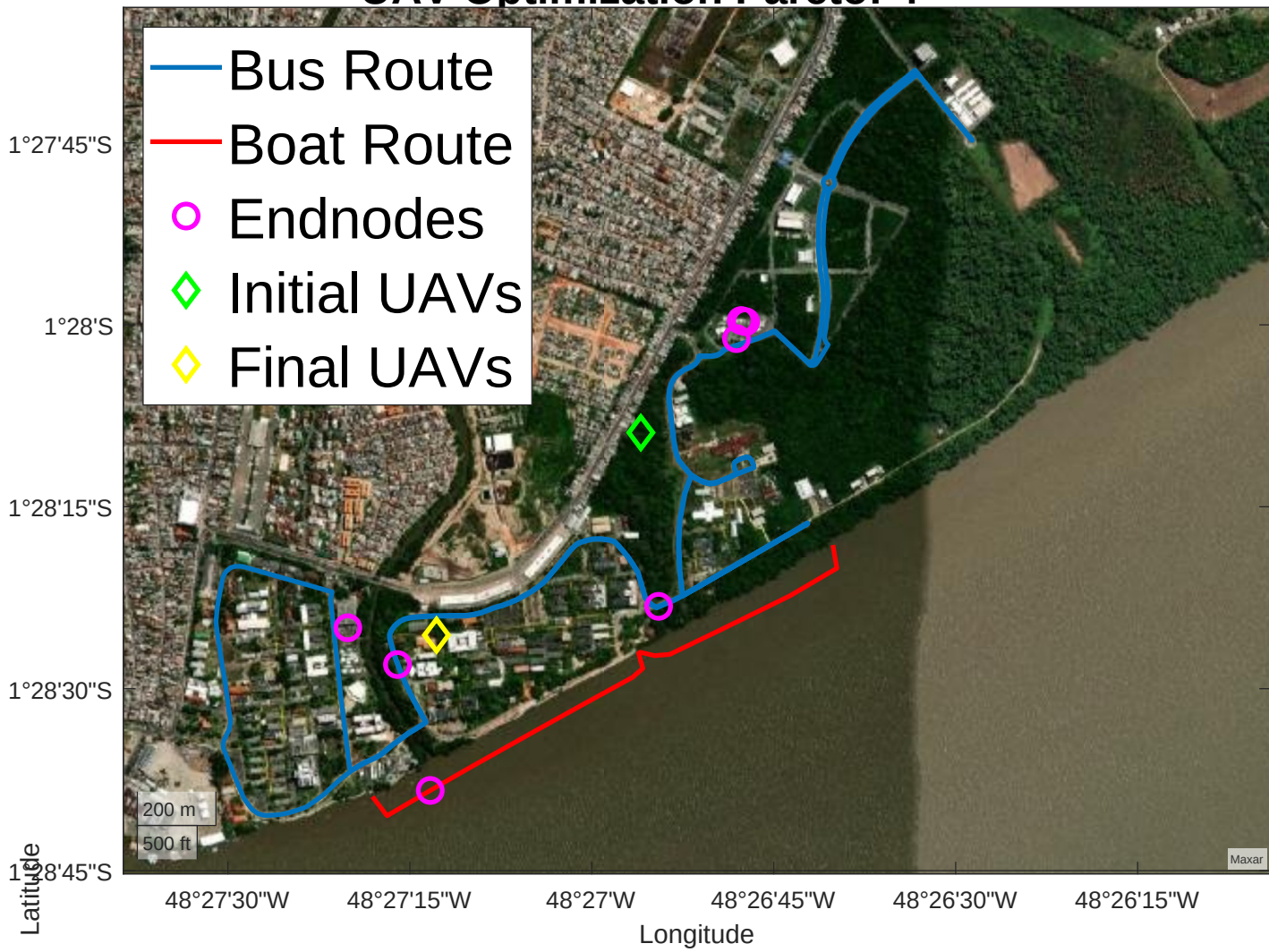

Supplement: Supplemental Information 13 [file peerj-cs-10-2237-s013.pdf]

# UAV Optimization Pareto: 5

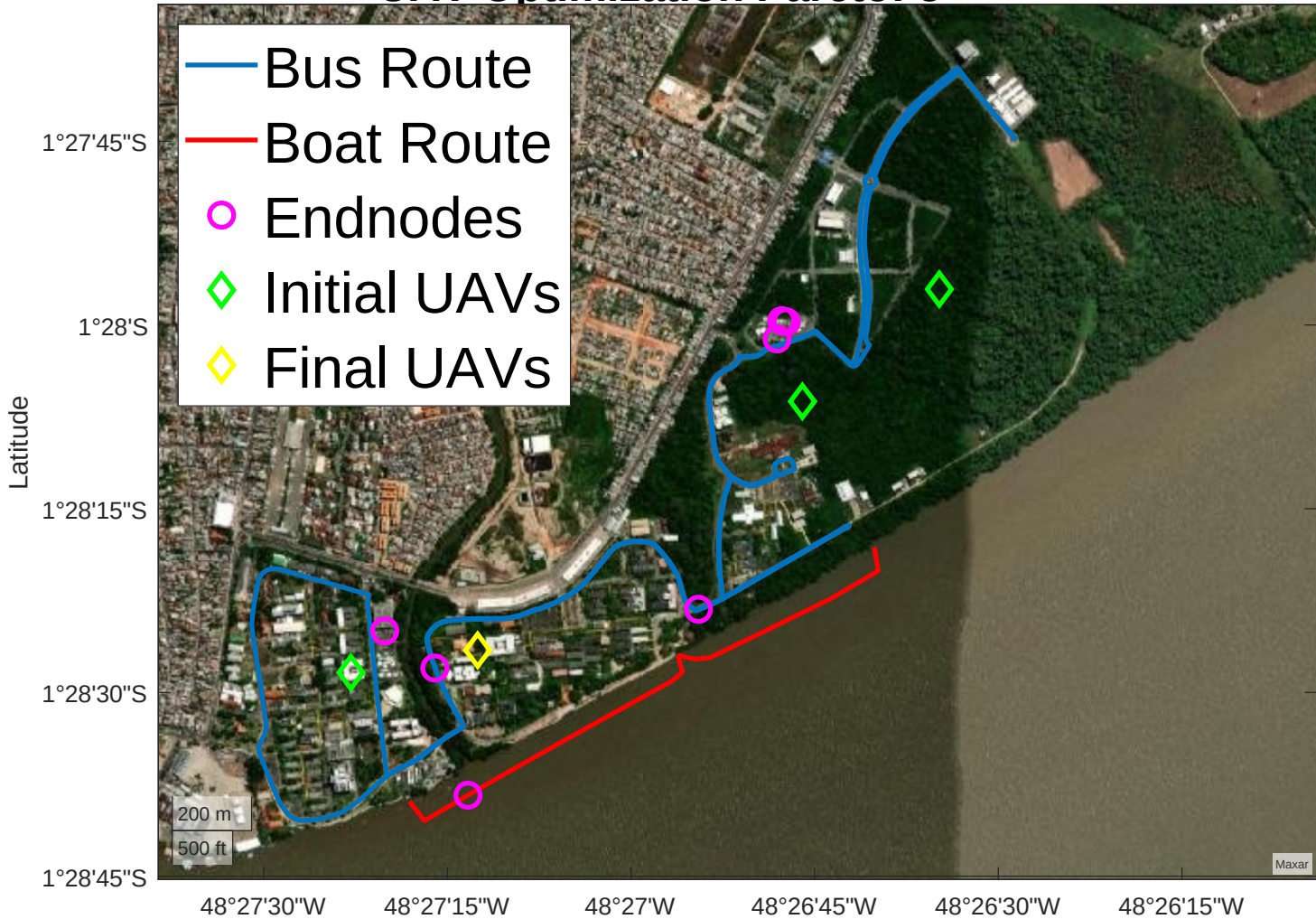

Supplement: Supplemental Information 14 [file peerj-cs-10-2237-s014.pdf]

# UAV Optimization Pareto: 6

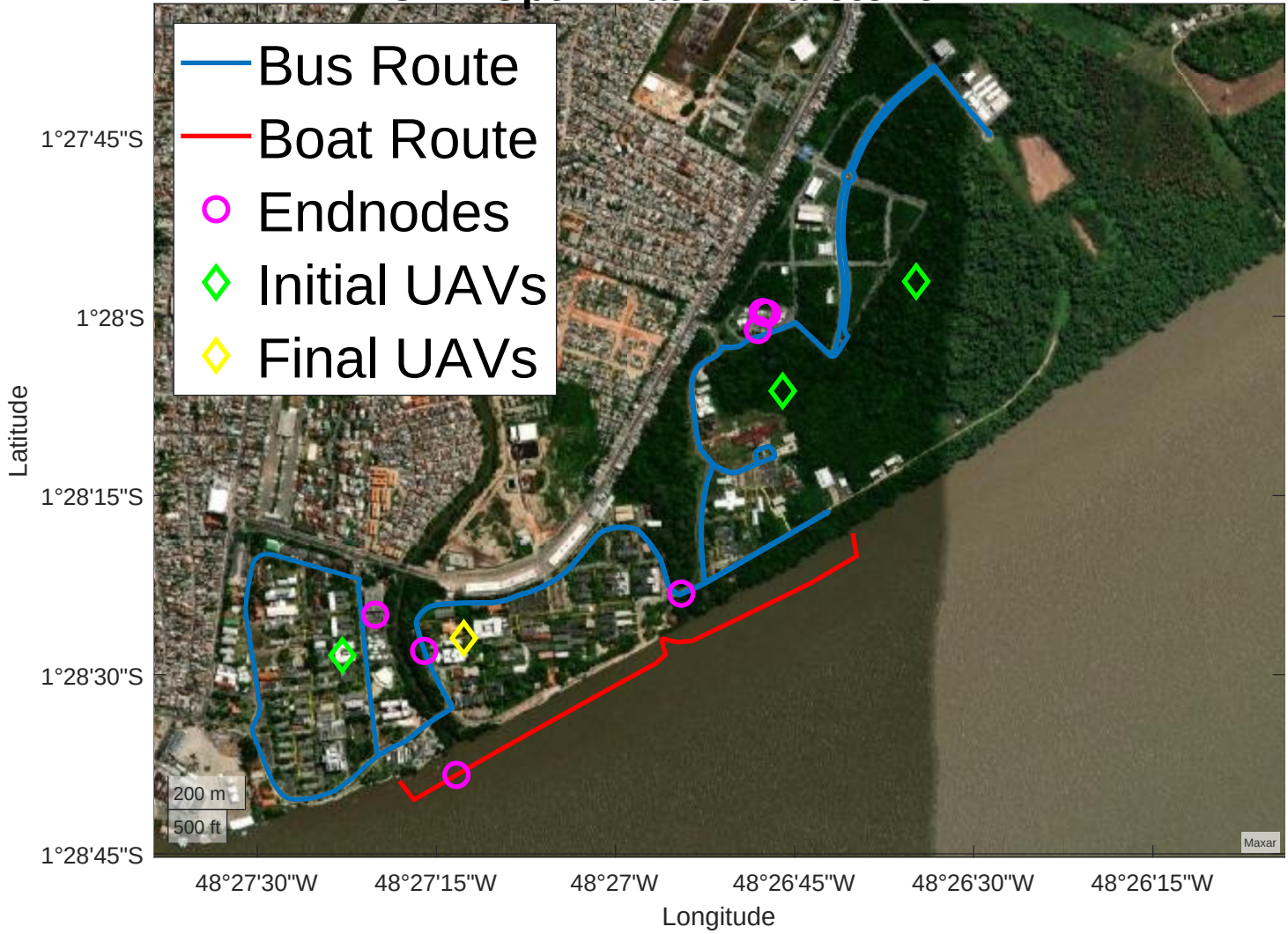

Supplement: Supplemental Information 15 [file peerj-cs-10-2237-s015.pdf]

# UAV Optimization Pareto: 9

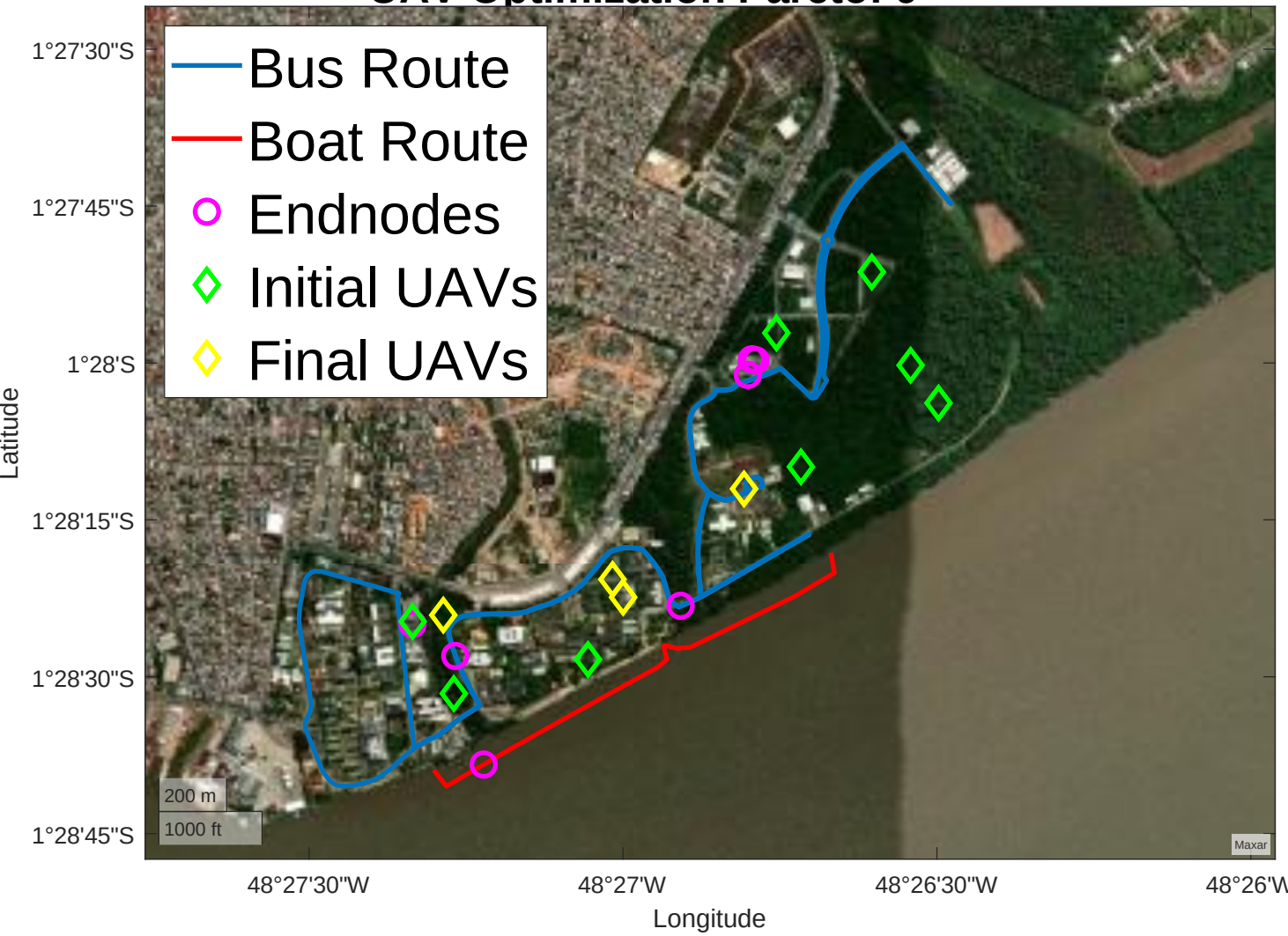

Supplement: Supplemental Information 16 [file peerj-cs-10-2237-s016.pdf]
